# Which is the proper reference tissue for measuring the change in FDG PET metabolic volume of cardiac sarcoidosis before and after steroid therapy?

**DOI:** 10.1186/s13550-018-0447-8

**Published:** 2018-10-05

**Authors:** Sho Furuya, Osamu Manabe, Hiroshi Ohira, Kenji Hirata, Tadao Aikawa, Masanao Naya, Ichizo Tsujino, Kazuhiro Koyanagawa, Toshihisa Anzai, Noriko Oyama-Manabe, Tohru Shiga

**Affiliations:** 1 Department of Nuclear Medicine, Hokkaido University Graduate School of Medicine, Kita 15 Nishi 7, Kita-Ku, Sapporo, Hokkaido 060-8638 Japan; 2 First Department of Medicine, Hokkaido University School of Medicine, Sapporo, Japan; 3 Department of Cardiovascular Medicine, Hokkaido University Hospital, Sapporo, Japan; 4 Department of Diagnostic and Interventional Radiology, Hokkaido University Hospital, Sapporo, Japan

**Keywords:** Cardiac sarcoidosis, ^18^F-Fluorodeoxyglucose, PET, Metabolic cardiac volume, Steroid therapy

## Abstract

**Background:**

Cardiac sarcoidosis (CS) is a rare but potentially life-threatening disease that causes conduction disturbance, systolic dysfunction, and, most notably, sudden cardiac death. ^18^F-fluorodeoxyglucose (FDG) positron emission tomography/computed tomography (PET/CT) plays important roles not only in diagnosing CS but also in evaluating the effects of anti-inflammatory therapy. A volume-based analysis of parameters measured by FDG PET, so-called cardiac metabolic volume (CMV), has emerged as a new assessment tool. CMV is measured as the volume within the boundary determined by a reference tissue such as the liver and the blood pool uptake. However, there is a possibility that oral steroid therapy could lead to variations of the liver and the blood pool uptake. Here, we attempted to evaluate the steroid effects on the liver and the blood pool uptake.

A total of 38 CS patients who underwent FDG PET/CT before and during steroid therapy were retrospectively enrolled. Volumes of interest (VOIs) were placed in the right lobe of the liver and descending aorta (DA). The maximum standardized uptake value (SUVmax), SUVmean, and SUVpeak of the liver and DA were compared between time points before and during steroid therapy.

**Results:**

The SUVmax, SUVmean, and SUVpeak of the liver during steroid therapy significantly increased from the time point before the therapy (SUVmax 3.5 ± 0.4 vs. 3.8 ± 0.6, *p* = 0.014; SUVmean 2.7 ± 0.3 vs. 3.0 ± 0.5, *p* = 0.0065; SUVpeak 3.0 ± 0.4 vs. 3.4 ± 0.6, *p* = 0.006). However, the SUVmax, SUVmean, and SUVpeak in the DA did not significantly change (SUVmax 2.2 ± 0.3 vs. 2.2 ± 0.4, *p* = 0.46; SUVmean 1.9 ± 0.3 vs. 2.0 ± 0.4, *p* = 0.56; SUVpeak 2.0 ± 0.3 vs. 2.0 ± 0.3, *p* = 0.70).

**Conclusions:**

We measured FDG uptake in the liver and blood pool before and during steroid therapy. Steroid therapy increased the liver uptake but not the blood pool uptake. Our findings suggested that the DA uptake is a more suitable threshold than liver uptake to evaluate therapeutic effects using volume-based analysis of cardiac FDG PET.

## Background

Sarcoidosis is a multisystem disease pathologically characterized by non-caseating granuloma [1–6]. Cardiac sarcoidosis (CS) occasionally causes cardiac death via congestive heart failure, ventricular tachyarrhythmia, and advanced conduction disturbance [1–5]. ^18^F-Fluorodeoxyglucose (FDG) is widely used as a positron emission tomography (PET) tracer to assess malignant and active inflammatory diseases. The main advantage of FDG PET is its ability to visualize metabolic activity to complement anatomic imaging [6, 7]. Previous studies have demonstrated the usefulness of FDG PET in diagnosing CS and monitoring treatment response [1–3].

In the context of FDG PET, SUVmax reflects only the value of a single voxel and does not reflect the metabolism of the entire target lesion. Volume-based parameters such as cardiac metabolic volume (CMV) or cardiac metabolic activity (CMA) have emerged as a novel measure, mainly for assessing the metabolism of CS [6, 8, 9]. CMV was defined as the volume of the cardiac FDG accumulation within a given boundary determined using a threshold such as the liver uptake, the blood pool SUV, and the fixed value of SUV [9–11].

Oral steroid therapy is the mainstay among treatment options for CS [1, 3]; however, the effect of steroids on background FDG uptake such as that of the liver and blood pool has never been evaluated. The aims of this study were (1) to assess any changes of FDG uptake in the liver and blood pool from time points before and during steroid therapy; (2) to examine the relationships between these reference uptakes and laboratory data such as fasting blood sugar (FBS), aspartate amino transferase (AST), alanine amino transferase (ALT), and γ-glutamyltranspeptidase (γ-GTP); and (3) to evaluate the association between the use of unfractionated heparin (UFH) and the reference FDG uptakes.

## Methods

### Study patients

This retrospective study was approved by the Institutional Review Board of Hokkaido University Hospital. Electronic medical records were reviewed to obtain laboratory test results. Between January 2010 and July 2017, 238 consecutive patients received an FDG PET/computed tomography (CT) scan for suspected or diagnosed CS. Patients with no abnormal FDG uptake in the heart (*n* = 153), patients with insufficient clinical evidence for medical intervention (*n* = 16), patients with abnormal FDG activity in the heart but who had started oral steroid treatment prior to the first PET scan (*n* = 8), patients who had not yet received a second PET scan (*n* = 4), patients who showed diffuse uptake in the liver (*n* = 4), and patients who were not treated with steroid therapy due to either mild symptoms (*n* = 3), initiation of steroid therapy in another hospital (*n* = 3), rejection of steroid therapy (*n* = 1), or diagnosis with malignant lymphoma (*n* = 1) were excluded. Seven patients were excluded simply because medical records were not available. Finally, 38 CS patients were included in the study. Laboratory testing was performed within an interval of less than 2 weeks of the PET scanning.

### Diagnosis

All patients met the widely accepted diagnostic criteria established by the Japanese Society of Sarcoidosis and Other Granulomatous Disorders (JSSOG) criteria or Heart Rhythm Society (HRS) consensus (Table 1) [12]. Diagnoses of CS were based on a combination of cardiac studies (electrocardiogram and cardiac ultrasound) and positive findings of FDG PET/CT with or without radiological studies including cardiac magnetic resonance imaging (MRI) and technetium-99m (^99m^Tc) myocardial perfusion imaging.

**Table 1:** Japanese Society of Sarcoidosis and Other Granulomatous Disorders (JSSOG) 2015 criteria for cardiac sarcoidosis

	Number of examined patients	Positive
JMHW criteria	38	38(100%)
Major criteria		
a. Advanced AV block	38	20(53%)
b. Basal thinning of the interventricular septum	38	21(55%)
c. Positive 18F-FDG uptake in the heart	38	38(100%)
d. Depressed EF (< 50%)	36	20(56%)
e. Gadolinium-enhanced CMR imaging	26	25(96%)
Minor criteria		
f. Abnormal ECG findings	38	22(58%)
g. Nuclear medicine: perfusion defect detected by myocardial scintigraphy	34	30(88%)
h. Endomyocardial biopsy: interstitial fibrosis	20	3(15%)

### FDG PET/CT imaging acquisition

FDG PET/CT data were acquired using a Biograph 64 True Point PET scanner with TrueV (Siemens Healthcare, Tokyo). Each patient fasted for at least 18 h before imaging. FBS levels were checked before the FDG injection. Approximately 4.5 MBq/kg of FDG was intravenously administrated under a resting condition. A static PET scan was performed 60 min after the administration of FDG. Intravenous preadministration of UFH was applied in the 25 patients at both the initial scan and the second scan: 5 patients only at the initial scan and 2 patients only at the second scan. Six patients did not receive a UFH injection at any scan.

The acquired datasets were corrected for attenuation by low-dose CT images and were reconstructed using a point-spread function-based iterative algorithm (TrueX; Siemens) with two iterations per 21 subsets, a matrix size of 168 × 168, a voxel size of 4.1 × 4.1 × 2.0 mm, and a Gaussian filter at 4.0 mm full width at half maximum. The transaxial and axial fields of view were 58.5 cm and 21.6 cm, respectively.

### Imaging analysis

We measured the SUVs in the liver by two methods as previously proposed: (1) a manual method with a 3-cm-diameter spherical VOI placed on the normal inferior right lobe (RL) of the liver [7] and (2) an automated method for objectively defining the liver VOI in FDG PET/CT [9]. With this automated method, the SUVmean and its standard deviation (SD) inside the VOI were used to determine the threshold value as follows: threshold = SUVmean + 3 × SD. SUVmax, SUVmean, and SUVpeak were estimated to compare the uptake before steroid therapy with that during the therapy. SUVpeak was defined as the average activity concentration within a 1-cm^3^ spherical VOI centered on the SUVmax voxel. The mean value of the Hounsfield units derived from CT was obtained using the same VOI. For the blood pool SUV, a 1-cm-diameter spherical VOI was set in the descending aorta (DA) so as not to overlap with the blood vessel wall as previously reported [13].

### Correlations between the FDG uptake and the preparation protocols

Our institute introduced dietary instructions using low-carbohydrate diet (LCHD) in 2010 to acquire sufficient suppression of physiological FDG uptake in the heart. Briefly, LCHD does not include bread or rice but include a boiled egg, tofu, and grilled chicken [4]. In this study, 23 patients were hospitalized and consumed a dinner < 5 g of carbohydrate the evening prior to FDG-PET scan. Fifteen patients were hospitalized and consumed a dinner not modified. Our institute stopped applying UFH before FDG injections in 2016, because it was deemed that UFH loading might suppress myocardial physiological uptake in patients with suspected CS while providing no extra diagnostic value compared with extended fasting [14]. As a result, our study included patients with different pre-scan protocols. (1) We compared the liver and DA uptakes between those with and without UFH administration to confirm the effect of UFH and, in addition, (2) we compared the liver and DA uptakes between patients with and without LCHD before FDG PET scans.

### Correlations between the liver uptake and clinical factors

Laboratory data such as serum AST, ALT, and γ-GTP, which are markers for liver dysfunction, were compared to the FDG uptake and Hounsfield units of the liver.

### Statistical analyses

Data are expressed as means ± standard deviations (SDs). Pairwise comparisons were performed with a paired *t* test. *p* values < 0.05 were considered to indicate statistical significance. Statistical calculations were carried out using SAS (JMP ver. 13; SAS, Cary, NC, USA). Multiple regression analysis was carried out to evaluate the effect of UFH on the liver and blood pool uptakes on the basis of patient factors, steroid therapy, and administrated UFH.

## Results

We identified 38 patients (61.4 ± 9.6 years old, 5 males) who presented with abnormal myocardial FDG uptake due to active CS out of a group of 238 consecutive patients. The clinical characteristics of the study subjects are shown in Table 2. Four patients were diagnosed with type 2 diabetes mellitus (T2DM). FBS before FDG injection was < 150 mg/dl for all patients, in both PET scans. None of the patients were rescheduled for the follow-up owing to a high BS level. The time span between the two scans and the amount of oral steroids at the second scan were 61.5 ± 23.5 days and 26.8 ± 4.3 mg/day, respectively.

**Table 2:** Characteristics of included patients

Factors	All patients (*n* = 38)
Age (years old)	61.4 ± 9.5
Gender (male)	5 (13.2%)
Diabetes mellitus	4 (10.5%)
BMI (kg/m^2^)	22.8 ± 3.6
Initial steroid dose (mg/day)	29.7 ± 1.6
Steroid dose at the second scan (mg/day)	26.8 ± 4.3
Duration between scans (days)	61.5 ± 23.5

*BMI* body mass index

### Differences in liver FDG uptake between pre- and mid-steroid therapies

Compared with the pre-steroid therapy scan, the liver thresholds during steroid therapy significantly increased in both the manual method (SUVmax 3.5 ± 0.4 vs. 3.8 ± 0.6, *p* = 0.014; SUVmean 2.7 ± 0.3 vs. 3.0 ± 0.5, *p* = 0.0065; SUVpeak 3.0 ± 0.4 vs. 3.4 ± 0.6, *p* = 0.0061) and the semi-automated method (SUVmean + 3SD; 3.5 ± 0.4 vs. 3.8 ± 0.6, *p* = 0.015).

**Fig. 1:**
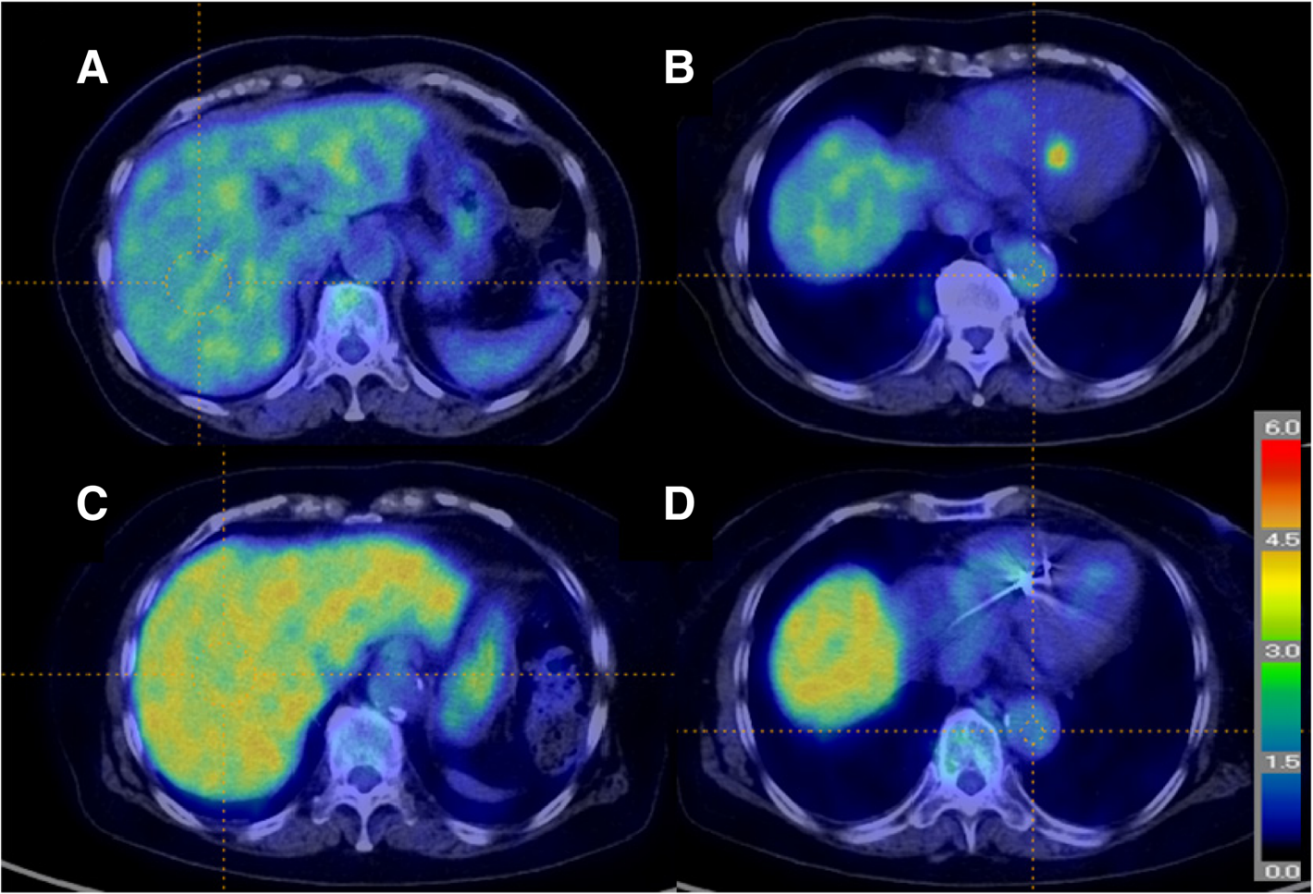
A representative case before and during the oral steroid therapy of CS. ^18^F-FDG PET/CT images obtained before (**a**, **b**) and during (**c**, **d**) treatment of CS. Note the profound decline in the myocardial uptake and increase in the liver uptake. The threshold increased from the time point before (**a**, 3.7) to that during steroid therapy (**c**, 4.5). The SUV of the DA showed little change from the time point before (**b**, SUVmax 2.9; SUVmean 2.6) to that during steroid therapy (**d**, SUVmax 2.8; SUVmean 2.5)

A typical case whose liver uptake was significantly increased during the oral steroid therapy is shown in Fig. 1.

### Differences in blood pool FDG uptakes between pre- and mid-steroid therapies

There were no significant differences in the SUVmax (2.2 ± 0.3 vs. 2.2 ± 0.4, *p* = 0.46), SUVmean (1.9 ± 0.3 vs. 2.0 ± 0.4, *p* = 0.56), or SUVpeak (2.0 ± 0.3 vs. 2.0 ± 0.3, *p* = 0.70) of the DA between the pre- and during-steroid therapy scans (Table 3). The SUV values assessed by the manual process in the DA did not show an association with the administrated UFH (Table 3).

**Table 3:** SUV change pre- to during-steroid therapy

	Region of threshold	Pre-therapy	During therapy	*p* value
Semi-automated method	Liver	3.5 ± 0.4	3.8 ± 0.6	0.02
SUVmax (manual process)	Liver	3.5 ± 0.4	3.8 ± 0.6	0.01
	DA	2.2 ± 0.3	2.2 ± 0.4	0.5
SUVmean (manual process)	Liver	2.7 ± 0.3	3.0 ± 0.5	0.007
	DA	1.9 ± 0.3	2.0 ± 0.4	0.6
SUVpeak (manual process)	Liver	3.0 ± 0.4	3.4 ± 0.6	0.006
	DA	2.0 ± 0.3	2.0 ± 0.3	0.7

*SUV* standardized uptake value, *DA* descending aorta

### Correlations between the FDG uptake and the preparation protocols

Before steroid therapy, 30 patients received UFH before FDG injection, whereas the remaining 8 patients did not. During steroid therapy, 27 patients received UFH before FDG injection, whereas the remaining 11 did not. The semi-automated thresholds of the liver were not associated with the use of UFH in patients before steroid therapy (Table 4). The SUVs in the liver during steroid therapy were significantly higher in patients in whom UFH was administrated (Table 4). However, multiple regression analysis showed no effect of UFH on the liver and blood pool uptakes. Both patient factors and steroid therapy had significant effects on the liver uptake (SUVmean + 3SD patient factor: *p* = 0.002, steroid therapy: *p* = 0.001, UFH: *p* = 0.54; SUVmax patient factor: *p* = 0.06, steroid therapy: *p* = 0.04, UFH: *p* = 0.46; SUVmean patient factor: *p* = 0.03, steroid therapy: *p* = 0.002, UFH: *p* = 0.75; SUVpeak patient factor: *p* = 0.02, steroid therapy: *p* = 0.001, UFH: *p* = 0.43). Only patient factor had significant effects on the blood pool uptake (SUVmax patient factor: *p* < 0.0001, steroid therapy: *p* = 0.07, UFH: *p* = 0.54; SUVmean patient factor: *p* < 0.0001, steroid therapy: *p* = 0.25, UFH: *p* = 0.36; SUVpeak patient factor: *p* < 0.0001, steroid therapy: *p* = 0.34, UFH: *p* = 0.88) (Table 5). Among 38 patients, 23 consumed LCHD in the evening both before and during steroid therapy, and the remaining 15 did not. Even when we divided the patients into those with and without LCHD, steroid therapy increased the liver uptake but not the blood pool uptake (Table 6).

**Table 4:** The effect of UFH for the thresholds: a paired *t* test

		Pre-therapy		*p* value	During therapy		*p* value
	Region	UFH (+) (*n* = 30)	UFH (−) (n = 8)		UFH (+) (*n* = 27)	UFH (−) (*n* = 11)	
SUVmean + 3SD	Liver 1	3.5 ± 0.4	3.6 ± 0.5	0.29	3.9 ± 0.6	3.4 ± 0.5	0.03
SUVmax	Liver 2	3.5 ± 0.4	3.6 ± 0.4	0.76	4.0 ± 0.6	3.5 ± 0.5	0.03
	DA	2.2 ± 0.3	2.1 ± 0.3	0.76	2.3 ± 0.3	2.1 ± 0.3	0.09
SUVmean	Liver 2	2.7 ± 0.3	2.8 ± 0.4	0.23	3.1 ± 0.5	2.7 ± 0.4	0.03
	DA	1.9 ± 0.3	1.9 ± 0.3	0.83	2.0 ± 0.3	1.8 ± 0.3	0.1
SUVpeak	Liver 2	3.0 ± 0.4	3.1 ± 0.4	0.61	3.5 ± 0.6	3.1 ± 0.5	0.03
	DA	2.0 ± 0.3	1.9 ± 0.3	0.51	2.0 ± 0.3	1.9 ± 0.3	0.06

*SD* standard deviation, *UFH* unfractionated heparin, *SUV* standardized uptake value, *Liver 1* SUV in the liver by semi-automated method, *Liver 2* SUV in the liver by manual process, *DA* descending aorta

**Table 5:** The effect of UFH for the thresholds: multiple regression analysis

		Patient				Steroid				UFH			
		Regression coefficient (95% CI)		*β*	*p* value	Regression coefficient (95% CI)		*β*	*p* value	Regression coefficient (95% CI)		*β*	*p* value
Liver uptake	SUVmean + 3SD	− 0.58	0.55	− 0.006	0.002	− 0.25	− 0.06	− 0.29	0.001	− 0.28	− 0.15	− 0.10	0.54
	SUVmax	− 0.51	0.49	− 0.005	0.06	− 0.22	− 0.05	0.32	0.04	− 0.22	0.16	− 0.06	0.46
	SUVpeak	− 0.62	0.60	− 0.004	0.02	− 0.27	− 0.008	− 0.33	0.001	− 0.32	0.14	− 0.15	0.43
	SUVmean	− 0.69	0.66	− 0.005	0.03	− 0.27	− 0.06	− 0.30	0.002	− 0.35	0.16	− 0.14	0.75
Blood pool uptake	SUVmax	− 0.20	0.20	− 0.007	< 0.0001	− 0.05	0.013	− 0.06	0.07	− 0.04	0.11	0.09	0.54
	SUVpeak	− 0.20	0.20	− 0.0003	< 0.0001	− 0.07	0.006	− 0.12	0.34	− 0.07	0.09	0.03	0.88
	SUVmean	− 0.19	0.19	− 0.0001	< 0.0001	− 0.05	0.016	− 0.05	0.25	− 0.08	0.07	0.05	0.36

*β* standard regression coefficient, *SUV* standardized uptake value

**Table 6:** The effect of low-carbohydrate diet for the thresholds

		Pre-therapy		*p* value	During therapy		*p* value
	Region	LCHD (+) (*n* = 23)	LCHD (−) (*n* = 15)		LCHD (+) (*n* = 23)	LCHD (−) (*n* = 15)	
SUVmean + 3SD	Liver 1	3.5 ± 0.4	3.5 ± 0.4	0.59	3.7 ± 0.7	4.0 ± 0.5	0.18
SUVmax	Liver 2	3.5 ± 0.4	3.5 ± 0.4	0.71	3.8 ± 0.7	3.9 ± 0.6	0.37
	DA	2.1 ± 0.3	2.2 ± 0.3	0.35	2.2 ± 0.4	2.3 ± 0.3	0.32
SUVmean	Liver 2	2.7 ± 0.3	2.7 ± 0.3	0.78	2.9 ± 0.5	3.1 ± 0.4	0.13
	DA	1.9 ± 0.3	2.0 ± 0.2	0.14	1.9 ± 0.4	2.1 ± 0.2	0.29

*SD* standard deviation, *LCHD* low-carbohydrate diet, *SUV* standardized uptake value, *Liver 1* SUV in the liver by semi-automated method, *Liver 2* SUV in the liver by manual process, *DA* descending aorta

### Correlations between liver uptake and each of the clinical factors and Hounsfield units

Compared to the values before steroid therapy, the FBS and serum ALT during steroid therapy were significantly higher. However, there were no significant differences in AST or γ-GTP between the initial and second scans (Table 7). Also, the mean values of Hounsfield units (HUs) were not significantly different between the two scans (49.9 ± 8.3 HU vs. 52.1 ± 6.8 HU, *p* = 0.22).

**Table 7:** Blood sample data

	First scan (pre-steroid therapy)	Second scan (during-steroid therapy)	*p* value
FBS (mg/dl)	90.8 ± 12.6	108.7 ± 22.6	< 0.0001
FDG dosage (MBq/kg)	4.4 ± 0.3	4.6 ± 0.5	0.10
AST (U/l)	24.0 ± 8.8	21.3 ± 8.4	0.19
ALT (U/l)	19.1 ± 10.1	33.1 ± 21.1	0.0004
γ-GTP (U/l)	30.8 ± 22.9	41.1 ± 24.0	0.06

*FBS* fasting blood sugar, *FDG* 18F-fluorodeoxyglucose, *AST* aspartate aminotransferase, *ALT* alanine aminotransferase, *γ-GTP* γ-glutamyltranspeptidase

## Discussion

In this study, we assessed the effect of steroid therapy on FDG uptake of the liver and DA by comparing PET images taken before and during steroid therapy. We assessed both the manual and automated methods of liver uptake analysis because several methods to set the VOI in the liver were reported. Wahl et al. proposed a manual method to set VOI on the normal inferior right lobe (RL) which is a widely used method in accordance with the Positron Emission Tomography Response Criteria in Solid Tumors (PERCIST) criteria [7]. Hirata et al. proposed an automated method to set the VOI in the liver, which was not always in the inferior RL, with very high inter-operator reproducibility [9]. Similar results were obtained by both methods.

The SUVs obtained from the liver during steroid therapy were significantly higher than those of the pre-therapy scan, whereas the SUVs from DA were not altered by steroid therapy.

CS is increasingly recognized as a cause of heart failure and arrhythmias. FDG PET is a promising tool to assess the activity of CS. The volume-based assessment of FDG uptake is a more precise predictor of cardiac events than SUVmax [1, 8]. An appropriate SUV threshold is important for the identification of the precise CMV. This study is the first to assess the effects of steroid therapy on the FDG thresholds used to estimate the metabolic volume.

CMV is defined as the volume within a given boundary determined using the FDG uptake threshold, such as the liver uptake and the blood pool [8, 10]. However, as our study suggested, the liver uptake in CS patients was significantly increased from the time point before to that during steroid therapy. Glucocorticoids promote changes in body composition that correlate with insulin resistance, hyperinsulinemia, and eventual onset of hyperglycemia [15–17]. Steroid-induced diabetes mellitus (SIDM) has been recognized as a complication of steroid use. The effect of glucocorticoids on glucose metabolism is likely the result of impairment of multiple pathways including sensitivity to glucose and the ability to release insulin due to the beta cell dysfunction and insulin resistance in other tissues [17]. One of the etiologies of SIDM is based on the effect of glyceroneogenesis in the liver and adipose tissue [18]. In the adipose tissue, glyceroneogenesis controls the rate of free fatty acid (FFA) release in the blood. On the other hand, glyceroneogenesis is responsible for the synthesis of triacylglycerol (TAG) from FFA and glycerol 3-phosphate (G-3-P) in the liver [18]. The regulation of this process in both the liver and adipose tissue occurs via the enzyme phosphoenolpyruvate carboxykinase (PEPCK). In patients using glucocorticosteroids, PEPCK gene expression in adipose tissue is suppressed, inhibiting glyceroneogenesis [19].

Because FDG uptake increases in the presence of inflammation, the increase of liver uptake may be due to an inflammatory process caused by steroid therapy [20, 21]. In the liver, PEPCK upregulates the synthesis of TAG from FFA and G-3-P, and liver fat increases. Although the HUs on CT did not show a significant difference, the accumulation of triglycerides in hepatocytes was assumed to have increased. While most fatty liver diffusely involves the whole liver, focal or multi-focal fat deposition in the liver is occasionally encountered and causes a diagnostic challenge. Some regions of the liver are well known as common sites of focal fat deposition [22, 23]. A 30-mm-diameter spherical VOI cannot assess the whole liver fat content and the CT HUs can be affected by focal fat deposition. [22].

Insulin resistance might be another potential reason for the increased liver uptake during steroid administration. In the liver, PEPCK stimulates glycerol production and FFA concentrations increase in the blood. In the end, the amount of FFAs released into the blood increases and the increased FFA level interferes with glucose utilization and results in insulin resistance, especially in skeletal muscle [24]. Though the specific effect of insulin on hepatic glucose uptake remains unclear, insulin stimulates glucose uptake in the liver of both insulin-sensitive and insulin-resistant subjects [25]. Iozzo et al., employing the euglycemic hyperinsulinemic clamp, confirmed that insulin increases hepatic phosphorylation of FDG to FDG-6-phosphatase (FDG-6-P) [25]. There is a strong evidence to suggest that hepatic steatosis and insulin resistances are driven by obesity-induced adipokines, and the association between insulin resistance and hepatic steatosis has been established [26, 27].

Although serum ALT levels are often used as a surrogate marker for liver inflammation, ALT is typically elevated in only 50% of non-alcoholic fatty liver disease (NAFLD) cases [20, 28]. In our patients, neither AST nor γ-GTP levels showed a statistically significant difference between time points before and during steroid therapy. Our results thus suggested that ALT alone was a poor marker for the presence of hepatic steatosis. Previous studies reported that patients with advanced fibrosis had significantly lower ALT levels than those with no/mild fibrosis and that ALT had no role in identifying patients with advanced disease [29, 30].

Our results showed that oral steroid therapy and individual patient factors showed significant effect on the blood pool and liver uptakes and UFH administration before FDG injection did not affect the liver or blood pool SUV [14]. UFH increases plasma FFA levels due to activation of lipoprotein and hepatic lipases [4]. As mentioned above, increased FFA interferes with glucose utilization and results in insulin resistance [24]. However, preadministration of UFH is just before FDG injection and the assessment of effect on liver uptake is difficult.

Oral steroid therapy showed no effect on the SUV obtained from the blood pool. Our group previously reported that individual FDG uptake thresholds from the DA were preferable to those from the liver, due to the high inter-operator reliability and non-dependence on dietary relations [13]. Our present findings show further evidence that the threshold for the evaluation of a therapy response should be determined by DA rather than liver values.

### Limitations

This study had some methodological limitations. First, the study was retrospective from a single center and the sample size was relatively small, partly because the study focus was patients with active cardiac sarcoidosis. Second, while all patients in our study underwent PET imaging, other tests, such as Holter monitoring and cardiac magnetic resonance imaging (MRI), were not routinely performed. Third, four patients with T2DM, who might have higher liver fat content compared with healthy subjects, were included in this study [26]. Fourth, we measured CT HUs using non-diagnostic low-dose CT as a tool of attenuation correction in PET/CT imaging. Compared to unenhanced diagnostic CT, a low-dose CT scan can be reliably used to exclude NAFLD, if neither liver attenuation of < 40 HU nor a liver-to-spleen ratio < 1.1 is present [31]. However, chemical shift images on MRI are desirable for the diagnosis of diffuse hepatic steatosis because they can demonstrate suppression of the signal from mixtures of microscopic lipids and water at the cellular level [20, 22]. Finally, we assessed the liver and the blood pool FDG uptake in patients using medium-dose steroids (29.7 ± 1.6 mg/day of prednisolone) but did not investigate whether the dose of the steroid affected the results. Oral steroid treatment for CS is generally initiated with 30 mg and is tapered down to 15–25 mg in most patients by the 3-month follow-up visit [1]. Further assessment of patients with tapered-down steroid use is warranted.

## Conclusions

We conducted a quantitative analysis of the liver and blood pool FDG uptake in patients diagnosed with CS. The liver FDG uptake was shown to significantly increase during steroid therapy. Thus, individual FDG uptake thresholds to assess the metabolic volume difference between time points before and during steroid therapy should be determined from the DA rather than liver uptake. Our results suggested that the DA is a more suitable threshold than liver uptake to evaluate cardiac metabolic volume.
